# Supplementary material for: Seminoma and Embryonal Carcinoma Footprints Identified by Analysis of Integrated Genome-Wide Epigenetic and Expression Profiles of Germ Cell Cancer Cell Lines
Source: PLoS One. 2014 Jun 2;9(6):e98330. doi: 10.1371/journal.pone.0098330 (PMC4041891; doi:10.1371/journal.pone.0098330)
Supplement: File S1 — ZIP file containing DMRforPairs output for significant regions. Please start from the html files. (ZIP) [file pone.0098330.s008.zip › figures/22322.pdf]

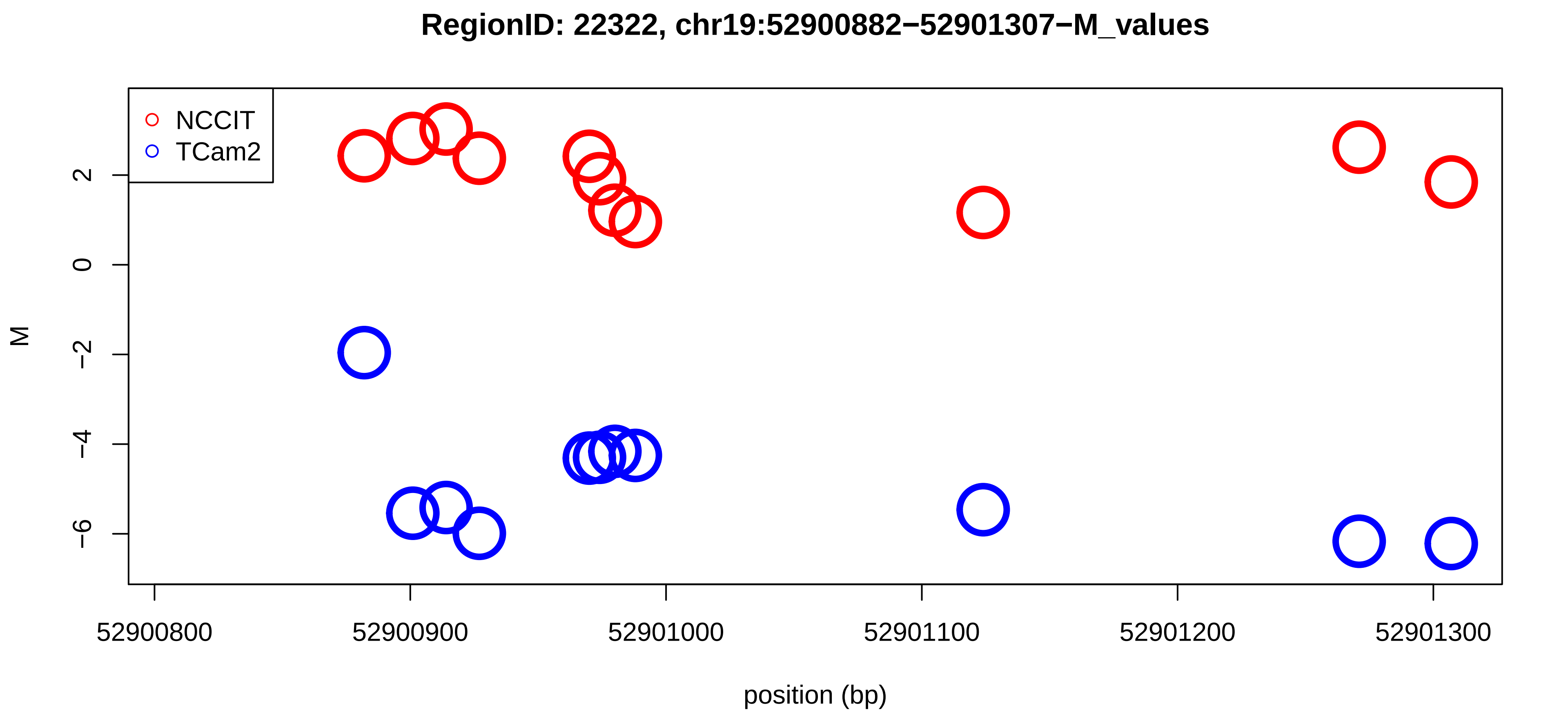
RegionID: 22322, chr19:52900882−52901307−M_values
NCCIT
TCam2
M
2
0
−2
−4
−6
52900800
52900900
52901000
52901100
52901200
52901300
position (bp)

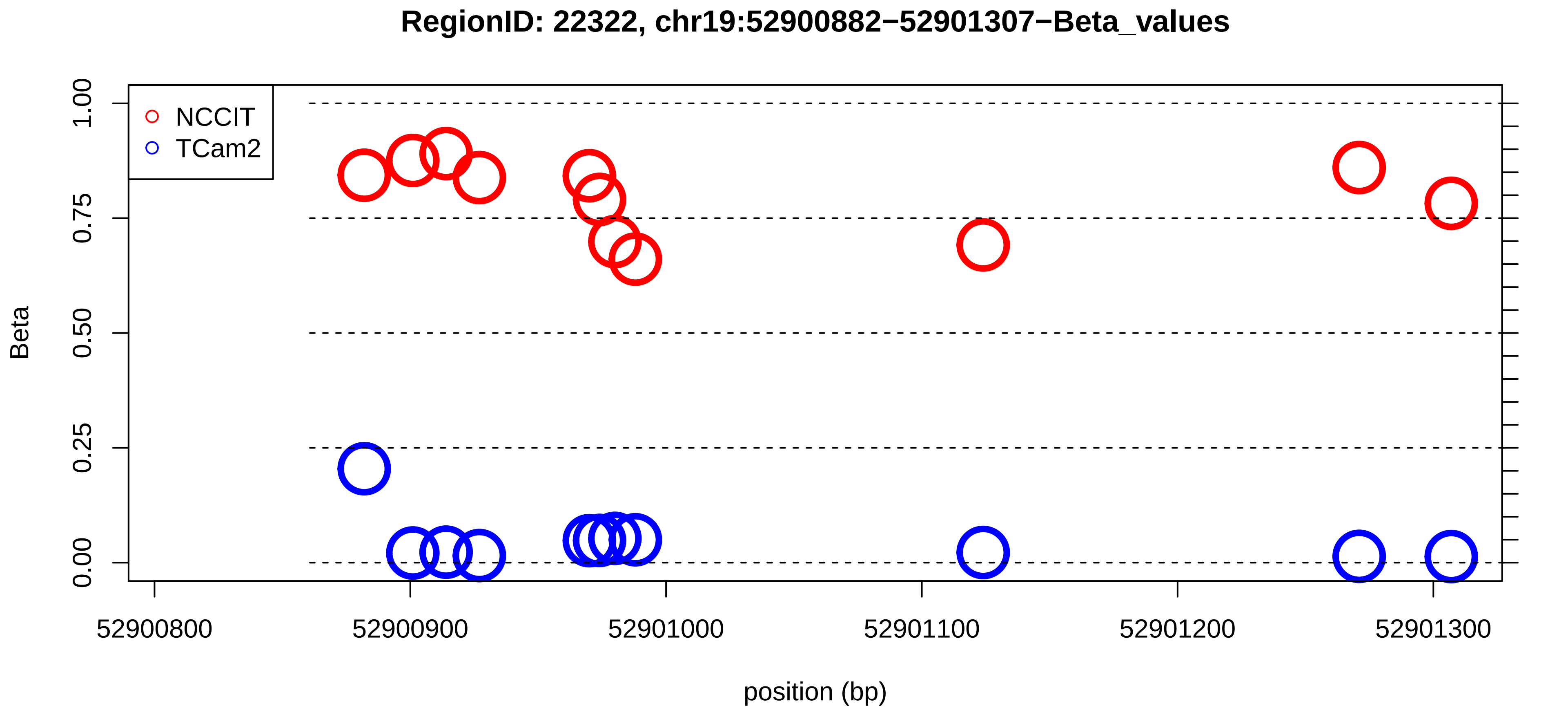
RegionID: 22322, chr19:52900882−52901307−Beta_values
NCCIT
TCam2
Beta
1.00
0.75
0.50
0.25
0.00
52900800
52900900
52901000
52901100
52901200
52901300
position (bp)

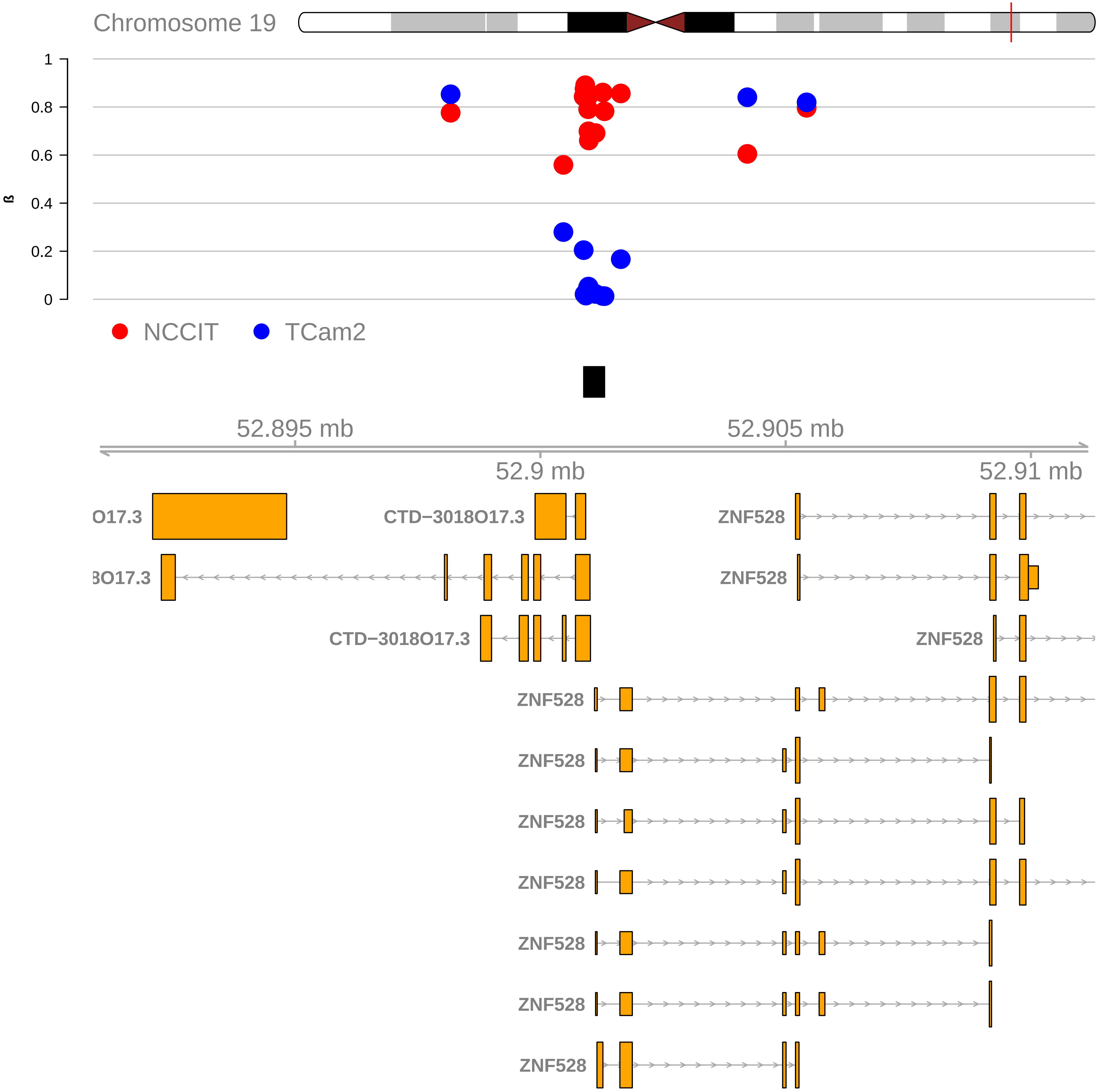

Chromosome 19
1
0.8
0.6
0.4
0.2
0
β
NCCIT
TCam2
52.895 mb
52.905 mb
52.9 mb
52.91 mb
O17.3
CTD−3018O17.3
ZNF528
3O17.3
ZNF528
CTD−3018O17.3
ZNF528
ZNF528
ZNF528
ZNF528
ZNF528
ZNF528
ZNF528
ZNF528
